# Experimental Protocol to Test Explicit Motor Learning–Cerebellar Theta Burst Stimulation

**DOI:** 10.3389/fresc.2021.720184

**Published:** 2021-09-08

**Authors:** Paola Ortelli, Davide Ferrazzoli, Roberto Maestri, Leopold Saltuari, Markus Kofler, Alessia Alibardi, Giacomo Koch, Danny Spampinato, Anna Castagna, Luca Sebastianelli, Viviana Versace

**Affiliations:** ^1^Department of Neurorehabilitation, Hospital of Vipiteno (SABES-ASDAA), Vipiteno-Sterzing, Italy; ^2^Istituto di Ricovero e Cura a Carattere Scientifico (IRCCS) Istituti Clinici Scientifici Maugeri, Pavia, Italy; ^3^Department of Neurology, Hochzirl Hospital, Zirl, Austria; ^4^Non-invasive Brain Stimulation Unit, Istituto di Ricovero e Cura a Carattere Scientifico (IRCCS) Santa Lucia Foundation, Rome, Italy; ^5^Department of Neuroscience and Rehabilitation, University of Ferrara, Ferrara, Italy; ^6^Department of Clinical and Movement Neurosciences, Institute of Neurology, University College London, London, United Kingdom; ^7^Istituto di Ricovero e Cura a Carattere Scientifico (IRCCS), Fondazione Don Carlo Gnocchi, Milan, Italy

**Keywords:** motor learning, cerebellum in motor learning, explicit motor sequential learning, motor adaptation, TMS, TBS

## Abstract

Implicit and explicit motor learning processes work interactively in everyday life to promote the creation of highly automatized motor behaviors. The cerebellum is crucial for motor sequence learning and adaptation, as it contributes to the error correction and to sensorimotor integration of on-going actions. A non-invasive cerebellar stimulation has been demonstrated to modulate implicit motor learning and adaptation. The present study aimed to explore the potential role of cerebellar theta burst stimulation (TBS) in modulating explicit motor learning and adaptation, in healthy subjects. Cerebellar TBS will be applied immediately before the learning phase of a computerized task based on a modified Serial Reaction Time Task (SRTT) paradigm. Here, we present a study protocol aimed at evaluating the behavioral effects of continuous (cTBS), intermittent TBS (iTBS), or sham Theta Burst Stimulation (TBS) on four different conditions: learning, adaptation, delayed recall and re-adaptation of SRTT. We are confident to find modulation of SRTT performance induced by cerebellar TBS, in particular, processing acceleration and reduction of error in all the conditions induced by cerebellar iTBS, as already known for implicit processes. On the other hand, we expect that cerebellar cTBS could induce opposite effects. Results from this protocol are supposed to advance the knowledge about the role of non-invasive cerebellar modulation in neurorehabilitation, providing clinicians with useful data for further exploiting this technique in different clinical conditions.

## Introduction

Motor learning can be defined as the set of processes, which allow creating internal abstract models of motor behavior. Each internal abstract model refers to a set of input-output relations between the generated (planned) motor commands and the subsequent environmental and internal consequences (1). These models entail two main components: the first one is defined by the spatial/sequential order in which movement modules are assembled into a defined action, the other one subserves action adaptation with respect to the individual and environmental needs (2–4). Learning how to ride a bicycle exemplifies the entire process, which entails different motor sequences to organize into a specific action schema: putting hands on handlebars, sitting on the saddle, positioning the feet on the pedals, applying muscle strength alternatingly on both legs, and propelling forward. These repetitive, modular motor sequences do not necessarily guarantee a good action outcome because of continuous adjustments, which depend on both the internal and external factors that need to be constantly adopted to fine-tune “on-going” actions (2, 4).

The motor learning process allows acquiring internal actions models through “allocentric” frames, and favors continuous adaptation to the specific needs and environmental circumstances using “egocentric” frames (5–7). Abstracted, goal-based, allocentric motor schemata are finally stored in the cortical-subcortical networks and enable the automatic performance of complex actions (8). Motor adaptation properly modulates these internal models of action based on egocentric, contextual, environmental, and ever-changing conditions for improving the action performance over time (4). In this context, one could think of how differently a bicycle ride might be by an inexpert amateur in comparison with a professional biker.

The internal models of motor behavior result from both implicit and explicit processes of learning (3, 9).

Differences between these two learning modalities do not account for the resulting internal abstract model, but refer to the generating process itself, which could be volitional and attention-demanding or conversely, unconscious, and non-attention based (9–11). The dissociation between procedural and declarative memory concerns the internal model and the distinctions between their verbally expressible and verbally inexpressible aspects (9, 12).

Motor learning results from activity in a complex cortico-subcortical network, which entails different areas, such as the motor cortex, the prefrontal cortex, the parietal cortex, the basal ganglia, and the cerebellum (1, 4, 13). Although the imaging data have so far not provided definitive evidence of a specific role of each area in the different phases of learning, consistent results indicate a crucial role of the cerebellum for motor sequence learning and motor adaptation, as it subserves error correction of on-going actions and contributes to sensorimotor integration (13–15).

Knowledge about motor learning has relevant, practical, and applicative values in neurorehabilitation, as principles of motor learning can be applied to the methodologies designed to promote clinical recovery in neurologically affected patients (16–18). The non-invasive brain stimulation techniques (NIBS), eventually combined with standard physical therapy (19–23), were shown to be effective in fostering and maximizing motor learning resources (24–29).

Augmentation of motor learning through the application of NIBS has previously been attempted in implicit motor learning paradigms (30–32). Indeed, modulation of the cerebellum was considered effective and easy, given the role this area plays in motor learning (33, 34).

The effect of a non-invasive cerebellar stimulation has been tested in various motor tasks (30, 35–44). A cerebellar repetitive transcranial magnetic stimulation (rTMS) induces long-lasting changes in the excitability of the contralateral primary motor cortex (M1) (45, 46) and may interfere with the cerebellar cognitive functions (47). Excitatory and inhibitory rTMS can also be administered in the form of TBS with the same efficacy and safety, but with the great advantage of requiring much shorter application times and lower stimulation intensities (48).

Recently, Koch et al. (49) demonstrated acceleration of motor adaptation following excitatory intermittent TBS (iTBS), applied to the right cerebellar hemisphere. Furthermore, TMS combined with electroencephalogram (TMS-EEG) revealed relevant changes of cortical activity in the interconnected motor networks induced by cerebellar iTBS (49). Block and Celnik (50) previously demonstrated enhancement of motor learning after anodal transcranial direct current stimulation (tDCS) of the right cerebellum but no effect in inter-manual transfer.

To date, the literature lacks studies exploring the explicit acquisition of allocentric motor sequences and the explicit, ego-centered adaptation of execution of the same sequences using different effectors (e.g., inter-manual transfer conditions). This is certainly a prominent aspect, considering the value and the effectiveness of explicit strategies in the field of neurorehabilitation.

To address explicit motor sequence learning, the Serial Reaction Time Task (SRTT) (5, 51, 52) was specifically designed (53). The participants are asked to perform a choice reaction time task, which consists of visual stimuli that appear on a screen in a fixed and repetitive sequence, and which requires moving specific parts of the body (usually fingers). It follows that the learning of a motor sequence will be implicitly or explicitly induced. Two variables, the reaction times and the accuracy of responses, permit analyzing both components of the learning process: acquisition of the movement sequence (the allocentric frame) and its adaptation (the ego-centric frame).

A study aimed to investigate the effects of cerebellar iTBS, continuous (cTBS), or sham TBS, in modulating both explicit motor sequential learning and motor adaptation in healthy subjects is presented.

## Materials and Methods

### Population

In this randomized, double blind experimental study, 40 healthy, right-handed participants were enrolled. Hemispheric dominance was assessed through the Edinburgh Handedness Inventory (EHI) (54). The inclusion criteria followed in the study were: (i) age 18–80 years, (ii) absence of visual or auditory disturbances possibly impacting on neuropsychological evaluation, (iii) no previous medical-neurological conditions considered as exclusion criteria for TMS (55), and (iv) no previous psychiatric diseases.

### Study Design

**Figure 1:**
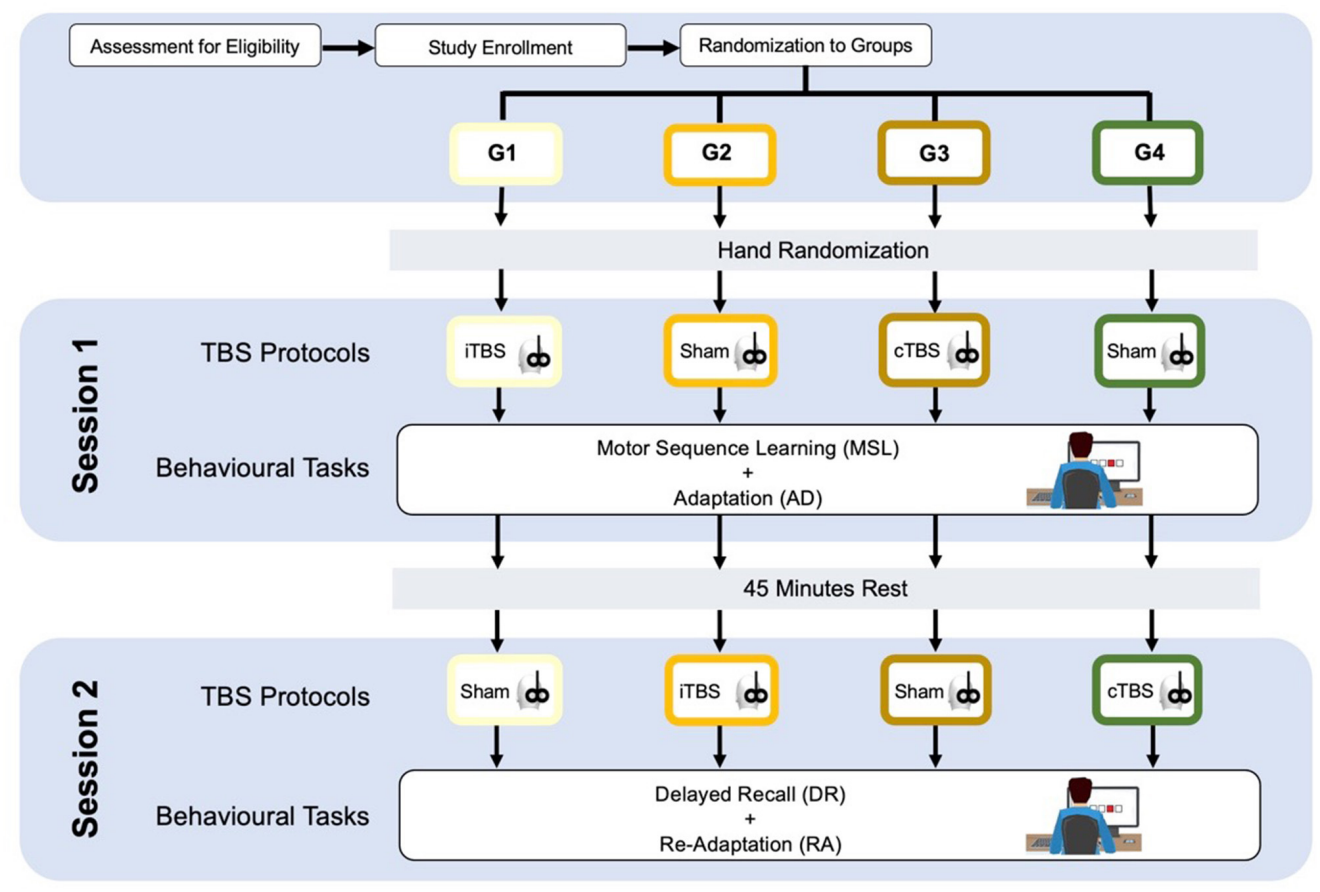
Study design. See the text.

The participants will be divided into four groups—age, sex, and educational-matched. All the participants will undergo two experimental sessions on a single-day, separated by a break of 45 min, each one including two behavioral tasks based on a modified SRTT paradigm (51–53). Before each session, patients will be subjected to one specific type of cerebellar TBS (Figure 1).

#### Theta Burst Stimulation

Group 1 (G1) will receive cerebellar iTBS before the session 1 and sham stimulation before the session 2; group 2 (G2) will receive cerebellar sham stimulation before the session 1 and cerebellar iTBS before the session 2; group 3 (G3) will receive cerebellar cTBS before the session 1 and sham stimulation before the session 2; group 4 (G4) will receive cerebellar sham stimulation before the session 1 and cerebellar cTBS before the session 2 (as shown in Figure 1).

Theta burst stimulation protocols will be carried out using a Magstim Super Rapid stimulator (Magstim Company, Whitland, UK) and a 70 mm figure-of-eight coil. TBS stimulation intensity will be set at 80% of the active motor threshold (AMT), defined as the lowest intensity which evoked at least five out of 10 MEPs with an amplitude > 200 μV peak-to-peak amplitude in the first dorsal interosseous (FDI) muscle during 10% of maximum voluntary contraction (56). This stimulation intensity was previously shown to produce plastic changes in the contralateral primary motor cortex (M1) (57). The iTBS protocol consists of a 2 s train of TBS repeated 20 times, every 10 s for a total of 190 s (48). The cTBS protocol consists of three-pulse bursts at 50 Hz repeated every 200 ms for 40 s (48). TBS will be applied over the lateral cerebellum, i.e., 1 cm inferior and 3 cm lateral to the inion (57). A cerebellar TBS is expected to modulate neural activity in the interconnected contralateral motor and parietal areas (57, 58). The coil will be positioned tangentially to the scalp, with the handle pointing superiorly; for sham TBS, it will be angled away so that no current was induced in the brain (59). Sham TBS will be delivered with an intermittent pattern in G1 and G2 and with continuous pattern in G3 and G4.

#### Behavioral Tasks

**Figure 2:**
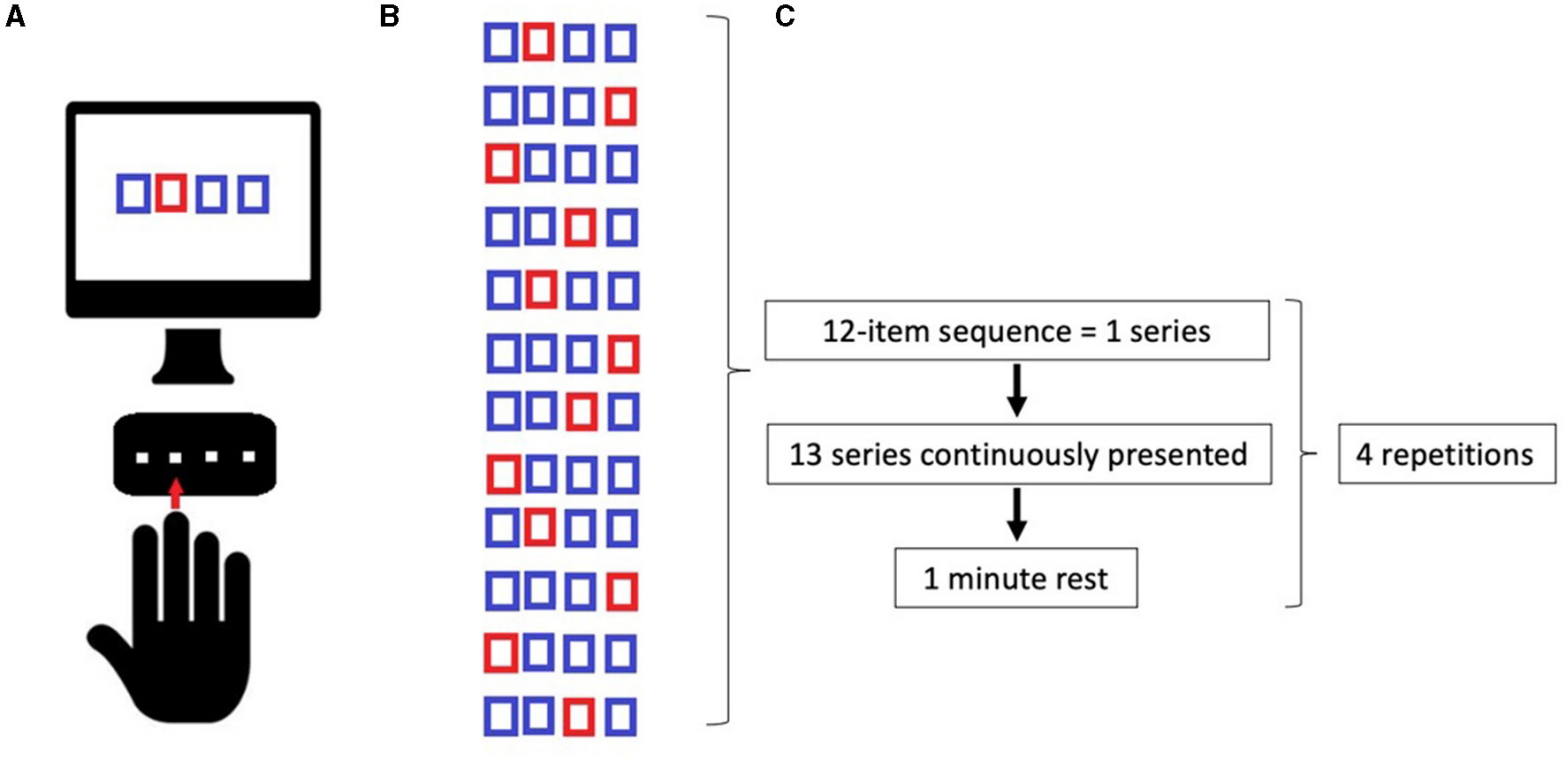
**(A)** Implementation of Serial Reaction Time Task (SRTT); **(B)** Learning sequence; **(C)** Timeline of tasks. See the text.

Serial Reaction Time Task was programmed using SuperLab 5 (Cedrus, San Pedro, CA, USA) and will be administered in a laboratory setting, with constant artificial light and without auditory interference. The hand to be used for the learning task will be randomized, and the contralateral hand subsequently will perform the adaptation task. The participants will seat on a chair in front of a computer screen, where four aligned blue squares will be presented simultaneously. As soon as one of these squares turns red (visual cue), participants are supposed to press as fast as possible the corresponding one of four aligned buttons on a keyboard with the corresponding index, middle, ring, or little finger (D2, D3, D4, and D5, respectively) of the selected hand. Immediately after correct key selection, the visual cue disappeared and the trial ends. After an interval of 200 ms, a new visual cue appeared and a new trial begins. Twelve consecutive trials correspond to the sequence that participants had to learn explicitly. The sequence needs to follow a specific set of rules: (a) the same visual cue does not appear two times successively; (b) each visual cue appears three times; and (c) visual cues are pseudo-randomized. The sequence to be learned is: 2–4–1–3–2–4–3–1–2–4–1–3. The same sequence will be used in each of the four experimental conditions: (1) Motor sequence learning (MSL), in which participants learn the sequence; (2) Adaptation (AD), in which participants are required to execute the learned sequence with the opposite hand; (3) Delayed recall (DR), in which the participants are required to recall the adapted sequence execution after a 45-min break; (4) Re-adaptation (RA), in which the participants have to return back to execute the sequence with the hand that originally learned the sequence (as shown in Figure 1). In MSL, participants will be required to learn the 12-item sequence presented in a 13-consecutive series, each series repeated four times with an inter-series-interval of 1 min. The same procedure will be repeated after 5 min. In AD, participants performed the same 13-consecutive series of 12-item sequences four times, with an inter-series-interval of 1 min with the opposite hand. In DR, participants repeated the AD task after 45 min and following another stimulation protocol (according to group assignment). Finally, in RA, participants will perform the 12-item sequence with the “original” hand in 13-consecutive series, will repeat four times with an inter-series-interval of 1 min. Errors, i.e., wrong key strokes, were counted (accuracy). The time taken to select the correct key will be recorded (response time, RT) (Figure 2).

The total protocol took maximum of 3 h with a 45-min break between the experimental sessions.

### Statistical Analysis

#### Behavioral Task Analysis

Shapiro–Wilk's statistic supported by visual inspection will be used to test the normality of distribution of all variables. Descriptive statistics of collected data will be reported as mean ± SD for continuous variables and as percentage frequencies for categorical variables. To assess data distribution, the variables will be examined by the Kolmogorov-Smirnov (K-S) test. One-way ANOVA will be used to compare differences in baseline values among groups. The effects of two independent variables (groups: specifically, G1 vs. G2; G3 vs. G4; G1 vs. G3; G2 vs. G4; task; MSL, DA, DR, and RA), on both accuracy and RTs of motor skill learning will be evaluated using two-way repeated measures ANOVA.

## Discussion

Motor learning is crucial for human behavior, from both a phylogenetic and ontogenetic point of view. To clarify the functional neural features underlying learning mechanisms, general explanations about plasticity have been widely provided. Nevertheless, although motor learning has been carefully investigated, many questions remain, to which often ambiguous and inconsistent answers are given. In particular, the distinction between implicit and explicit learning is occasionally equivocal and could generate misunderstandings when applied to the concept of movement. From a classical point of view, implicit learning refers to procedural and unconsciousness knowledge, whereas explicit learning refers to declarative and conscious knowledge. More recently, Keele et al. (11) proposed a model in which the dichotomy inherent to the distinction between implicit and explicit learning modalities refers to the involvement of attentional resources in the learning process (11). In their perspective, implicit learning requires a low-level of attentional demand, while explicit learning refers to the need for substantially more attentional resources, thus implying a continuous rather than dichotomous nature of the two learning modalities, and indeed, previous data revealed an overlap of these two, both in terms of neural processing and of involved cerebral networks (9). This concept concurs well with the so-called “hodotopic” organization of the central nervous system in parallel, dynamic, and interactive networks: thus, human functions, such as learning and memory, have to be re-thought beyond their modular and rigid conceptualization. Otherwise, it does not imply a loss in their functional specificity, which remains absolutely crucial from both research and applicative perspectives (9, 60).

The existence of an interactive overlap among different learning processes could be considered as a point of strength in human motor behavior. Moreover, from a neurorehabilitation point of view, the distinction between implicit and explicit strategies to be applied in the clinical context, and the comprehension of their role in motor learning and adaption are pivotal.

The acquisition and improvement of motor skills are one of the central goals in neurorehabilitation. However, circumstances, in which motor learning strategies are applied may differ substantially from patient to patient and depend on the clinical situation (61). Actually, there is no consensus regarding the “correct” application of the implicit and the explicit motor learning strategies in neurorehabilitation. An explicit motor learning approach, based on volition and attention, makes use of explicit verbal and/or visual instructions, strategies to be applied to reach a given goal, cues, and feedback. These rules are not addressable for implicit motor learning, given its proper nature: implicit learners may be able to free-up attentional resources to perform given tasks (62). Going beyond this distinction, it is important to understand that the application of motor learning strategies has to be set with respect to the functional state of the patients (63–65). Therefore, while implicit learning may be particularly beneficial for those patients who suffer from cognitive impairments (e.g., amnesia or aphasic), others may particularly benefit from receiving explicit explanations (e.g., in patients with Parkinson's disease).

To bridge the gap in the field, we have designed an experimental study that aimed at evaluating whether a non-invasive stimulation of the cerebellum affects performances related to explicit motor learning and adaptation. Due to the cross-sectional design of the study, we expect to be able to distinguish between the encoding and retrieval phases.

The cerebellum is a unique hub in the central nervous system, as it is phylogenetically developed for receiving and integrating both afferent and efferent inputs from almost the entire brain. Its structural organization promotes the integration of motor and non-motor aspects of behavior and their subsequent predictive computation, which is required for motor learning and motor control (66, 67). The role of the cerebellum in implicit learning has been extensively studied (49, 68), whereas little is known about its possible role in explicit learning (3, 69).

We expect to confirm the previously described findings (49), which showed improvement of implicit motor adaptation and subsequent re-adaptation following cerebellar iTBS (49). Furthermore, we anticipate that iTBS will positively affect all phases of explicit motor learning and adaptation by enhancing the predictive computation in favor of voluntary-engaged attentional strategies. Conversely, cerebellar cTBS could induce the opposite effect.

In the future, results from this study could be translated in the clinical setting for implementing and extending the use of NIBS in neurorehabilitation. In this perspective, the effectiveness of rehabilitation strategies to be applied in all the phases of the re-learning process could be hopefully empowered.

## Data Availability Statement

The original contributions presented in the study are included in the article/supplementary files, further inquiries can be directed to the corresponding author.

## Author Contributions

PO, DF, AA, and VV wrote the manuscript. PO, DF, DS, LSa, LSe, MK, and VV designed the study and gave substantial intellectual contribution to the manuscript content. RM statistical support. All authors contributed to the article and approved the submitted version.

## Conflict of Interest

The authors declare that the research was conducted in the absence of any commercial or financial relationships that could be construed as a potential conflict of interest.

## Publisher's Note

All claims expressed in this article are solely those of the authors and do not necessarily represent those of their affiliated organizations, or those of the publisher, the editors and the reviewers. Any product that may be evaluated in this article, or claim that may be made by its manufacturer, is not guaranteed or endorsed by the publisher.
